# CDP++.Italian: Modelling Sublexical and Supralexical Inconsistency in a Shallow Orthography

**DOI:** 10.1371/journal.pone.0094291

**Published:** 2014-04-16

**Authors:** Conrad Perry, Johannes C. Ziegler, Marco Zorzi

**Affiliations:** 1 Faculty of Life and Social Sciences, Swinburne University of Technology, Hawthorn, Australia; 2 Aix-Marseille Université and Centre National de la Recherche Scientifique, Marseille, France; 3 Dipartimento di Psicologia Generale and Center for Cognitive Neuroscience, Università di Padova, Padova, Italy; 4 IRCCS San Camillo Neurorehabilitation Hospital, Lido di Venezia, Italy; The National Institutes of Health, United States of America

## Abstract

Most models of reading aloud have been constructed to explain data in relatively complex orthographies like English and French. Here, we created an Italian version of the Connectionist Dual Process Model of Reading Aloud (CDP++) to examine the extent to which the model could predict data in a language which has relatively simple orthography-phonology relationships but is relatively complex at a suprasegmental (word stress) level. We show that the model exhibits good quantitative performance and accounts for key phenomena observed in naming studies, including some apparently contradictory findings. These effects include stress regularity and stress consistency, both of which have been especially important in studies of word recognition and reading aloud in Italian. Overall, the results of the model compare favourably to an alternative connectionist model that can learn non-linear spelling-to-sound mappings. This suggests that CDP++ is currently the leading computational model of reading aloud in Italian, and that its simple linear learning mechanism adequately captures the statistical regularities of the spelling-to-sound mapping both at the segmental and supra-segmental levels.

## Introduction

The way orthographies represent sound differs markedly across languages. English, for example, is generally thought to have a comparatively complex orthography (e.g., [1]). One promising strategy to investigate how differences across orthographies may shape the functional architecture of the reading system is to develop full-blown computational models of reading for different languages using a common framework and the same core processing components (e.g., [2], [3]). This strategy is pursued here in the context of the Connectionist Dual Process Model of Reading Aloud (CDP) [4]–[11], a model that was originally developed for English. The latest versions of this model (e.g., CDP++ [5]) have been shown to provide the most comprehensive account of the empirical data, outperforming all of their competitors by an order of magnitude in terms of quantitative performance (i.e., goodness of fit).

Unlike English, Italian is characterized by relatively simple (i.e., transparent) relationships between orthography and phonology. It therefore provides an interesting contrast with respect to the bulk of research on the far less transparent English orthography (see e.g., [12] for a discussion). As is often the case, simplicity at one level comes at the price of complexity at another level. Finnish is a good example of this, where grapheme-phoneme relationships are extremely simple (fully consistent) but the morphological system is highly complex. In Italian, complexity can be found at the suprasegmental (word stress) level (e.g., [13]). That is, words with the same syllable structure and similar spellings can have stress in different locations (syllables) and where stress goes is not always predictable from the sublexical information. For example, in the database that is used below, 77.2% of 3-syllable words have stress on the penultimate syllable (e.g., *do'mani* [tomorrow]), 13.2% have stress on the antepenultimate syllable (e.g., '*macchina* [machine]), and 9.6% have stress on the ultimate syllable (e.g., *socie'tà* [society/group]).

Apart from Italian stress being interesting in its own right, it is interesting to compare stress assignment in English and Italian as this comparison makes it possible to investigate whether the same mechanism can be used to assign stress in languages that differ in terms of complexity. Cross-language differences between English and Italian show that not only are the linguistic “rules” of how stress is assigned quite different (cf., e.g., [13] and [14]), but so are other factors. These include morphology (c.f., [13] and [15]) and orthographic markers that help predict stress (e.g., [16]–[18]). For example, with orthographic markers, Italian uses a diacritic (*à*) to mark stress in word-final position which English does not commonly use and both languages have orthographic sequences that are correlated with certain stress patterns. The existence of different cues that may have different weights in different languages is a challenge for a model like CDP++ because it uses the same architecture and learning mechanisms in different languages. Thus, the model needs to find the “right” cues solely on the basis of the statistical spelling-to-sound properties in the training corpus. Similarly, if there are patterns in the data that are seemingly best accounted for by a rule system, such as that which has been suggested for predicting the stress of Italian verbs [13], CDP++ and other connectionist models (e.g., [5], [6], [19], [20]) must learn to approximate these patterns without the use of a rule-based mechanism.

There are now a fairly large number of published studies that have investigated different aspects of word and nonword reading in Italian, many of which are specifically concerned with how stress is computed. This means that it is possible to thoroughly test computational models of Italian reading aloud [12]. A key prediction of the CDP approach is that the relationships between spelling and sound as well as spelling and word stress can be learnt via a simple linear learning mechanism. Given the complexity of word stress in Italian, it remains a challenging question whether such a simple mechanism can correctly predict stress assignment along with a number of other effects that have been reported. We therefore constructed an Italian version of CDP++ and assessed its descriptive adequacy both qualitatively and quantitatively.

In terms of the scope of data to test the model on, we focused on skilled adult reading. We selected all studies that used a simple naming or priming paradigm with literate adults, for which the authors provided the list of items used. Only those studies that used more than 12 items in each cell were chosen. We also examined the largest study that investigated the effect of stress in acquired dyslexia in Italian [21]. Developmental studies were not used to test the model because there are a number of issues to do with development that make simulating these data beyond the scope of the current work (for a discussion of these issues and a developmental CDP++ model, see [22]–[24]).

### The Model: CDP++.Italian

**Figure 1:**
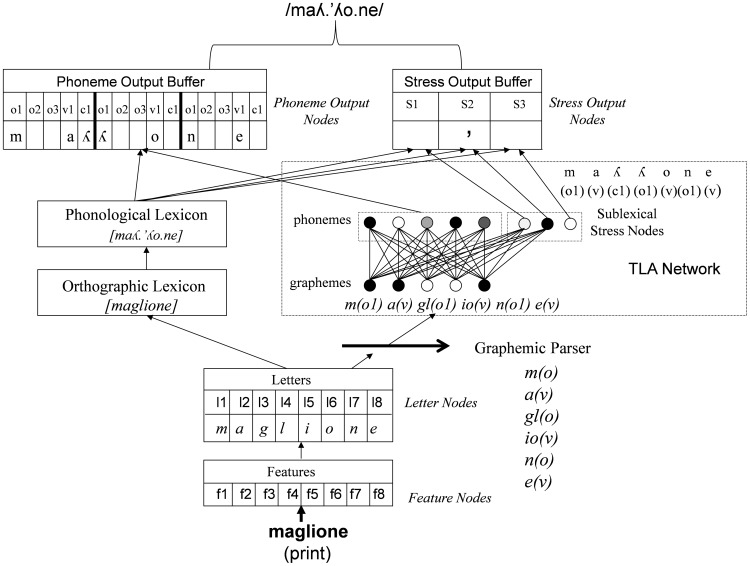
CDP++.Italian. Note f = feature, l = letter, S = Stress, o = onset, v = vowel, c = coda. Numbers correspond to the overall slot number with the letter and feature nodes or the particular slot within an onset, vowel, or coda grouping for the rest of the representations. The thick divisors in the Phoneme Output Buffer represent syllable boundaries. The grapheme and phoneme nodes in the TLA network are simply used as an example, and do not correspond to the actual set of graphemes used in the network.

The architecture and the processing assumptions of the model are identical to those of the latest version of the English CDP++ [6], except that rather than only using words with a maximum of two syllables, words with three syllables were also used. In line with its dual-process framework, the model includes two main routes between spelling and sound, a lexical and a sublexical route (see Figure 1). The lexical route is identical to all versions of CDP (apart from the earliest version), excluding the database used and its properties. The new database (see below) meant that 35 phonemes used and 32 letters (including a null letter) were used. The feature level of the model contained 14 features, although the parameters were set so that feature overlap had essentially no effect on the performance of the model in any way. In terms of the other parts of the lexical route of the model, the same frequency counts were used in both the orthographic and phonological lexicons since the database we used only had one set of frequency counts. In addition, all of the words used a frequency count that was the same as those given in the database plus 2. This was done because some words have a frequency value of zero and we take log values of frequencies for some computations. This means all frequency values always end up being greater than 0.

The sublexical route consists of a graphemic parser and a two-layer associative (TLA) network. The graphemic parser is designed to segment letter strings into graphemes as well as categorize the graphemes into onset, vowel, and coda categories. This categorization process allows the graphemes to be placed into the graphosyllabic template of the TLA network (i.e., its input representation), and the TLA network is then able to generate phonology from them. The two different routes converge at the phoneme output buffer, where phonemic activation is integrated, as well as at the stress output buffer, where word stress activation is integrated.

**Figure 2:**
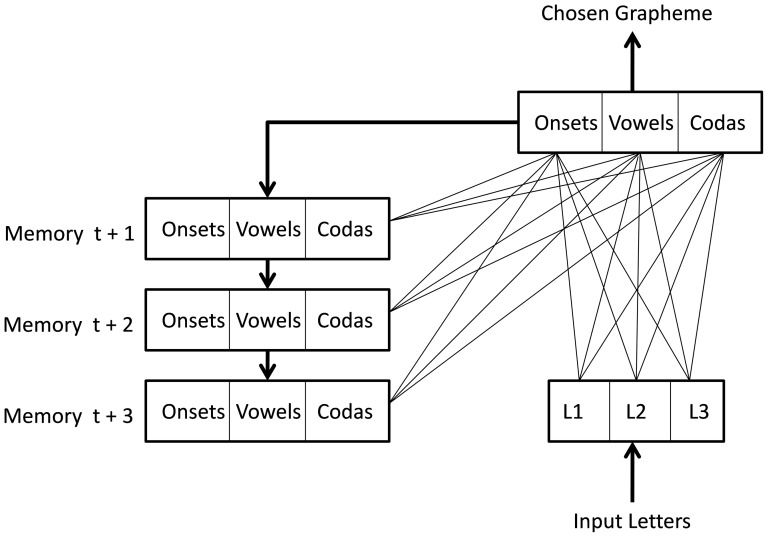
The graphemic parser. Note: t = time; L = Letter.

At present, learning only occurs in the sublexical route when the model is in training mode. In this mode, the graphemic parser is presented with a set of letter strings generated from each word. These strings are constructed based on the idea that an *attentional window* moves over letter strings from left to right, with the model learning which grapheme is at the start of each letter string within the attentional window (i.e., a set of letters is presented and the parser produces a grapheme that can be one or more letters long as an output). Apart from just learning which grapheme is at the start of a string, the graphemic parser also learns what type of grapheme it is (onset, vowel, or coda). In running mode, this allows the graphemes to be placed in a syllable-like template (i.e., the graphosyllabic template of the TLA network) based on their categorization, since if an onset grapheme follows a vowel or a coda grapheme, it means that it must be placed into the next syllable of the template after the vowel. Finally, the parsing network has a memory for previous graphemes it has parsed. This allows some amount of context sensitivity to be learnt even when the letters in the attentional window are the same, which is important for languages like English (see [6]). The parser is displayed in Figure 2.

In Italian, because the correspondences between spelling and sound are less complex and have less contextual sensitivity compared to English, the attentional window is only 3 letters wide (in English, we used 5). This means that with the word *maglione* [jumper], for example, six strings of letters would be given to the network as input patterns: mag, agl, gli, ion, ne*, e** (note that the * represents no letter is in the window). The teaching signal (i.e., desired output) presented to the network during learning is the first grapheme occurring in each letter string, as well as its category (for the example above: m, onset; a, vowel; gl, onset; etc.). The three categories are represented in the output by simply duplicating the set of graphemes three times, and the grapheme is put in the set that represents the category it belongs to. Once the network has learnt relationships between the letter strings and the first grapheme in the strings, the parser can break strings of any length down into graphemes as well as place them into their correct position in the graphemic buffer. Thus, in running mode, the constituent graphemes for any string of letters (regardless of whether it is a known word, a novel word, or a nonword) are generated in an entirely bottom-up fashion.

The orthography-to-phonology mapping is learnt by the TLA network, which is presented with graphemes (inputs) and, during learning, phonemes and stress information (outputs). In learning mode, all of the information is presented at the same time, and the model learns simple associations between inputs and outputs using the delta rule (this is equivalent to the Rescorla-Wagner learning rule [25]). In running mode, the graphemes are placed in the graphemic buffer of the TLA network in a position determined by the graphemic parser, and the model generates phonology and stress information based on the simple associations it has learned during training. The information from the output of this network is then used in conjunction with activation produced by the lexical network to generate a pronunciation.

### Database

The lexicon of the model was constructed from all words up to 3 syllables and 8 letters long that were in the Adsett et al. [26] database (N = 63,438). This database consists of a large number of Italian words, and morphologically simple and morphologically complex word forms are represented separately. Letters in the database with a diacritic (accent) mark were coded as an entirely separate letter, which meant there were 31 separate letters plus one for the ‘blank’ letter. In terms of phonology, stress was coded based on syllable position (i.e., 1^st^, 2^nd^, or 3^rd^), and there were 32 phonemes in the database, of which 23 were consonants, 7 were vowels, and 2 were semi-vowels. Frequency counts were obtained by entering the words into the Google search page on the 15/8/2008 and counting the hits for each word, with an Italian language restriction. Whilst it is known that Google counts may not be perfect [27], the log frequencies of the counts correlated reasonably well with the log frequencies in the CoLFIS database [28] using all items that were shared, r = .77 (N = 21279). In addition, when frequency alone was used as a predictor on the Barca et al. [29] database of written word naming latencies, an almost identical sized correlation (r = −.24) was found with both the CoLFIS frequencies and Google counts. There are a number of very low frequency words in the Adsett et al. database that are unlikely to be known by most of the Italian speaking population, as well as a number of loan words. These words were left in the database for the sake of simplicity and generality. There is also a reasonable amount of variation in different Italian dialects, and the examples used here are taken directly from the Adsett et al. database, and thus may differ as a function of regional dialects.

### Graphosyllabic Template

The basic idea of the graphosyllabic template is to allow graphemes to be put into a syllable-like structure. In learning mode, where exemplars are presented to the model and the structure is learnt, this graphemic structure is derived by trying to align graphemes with lexical phonology, although other methods could certainly be used (see [6] for a discussion). This means that identical letter sequences can potentially be coded differently if those sequences map to different lexical phonology. To code these sequences, a number of assumptions were made about graphemes and how they are placed in the graphosyllabic template.

First, in terms of the set of multi-letter graphemes, these were selected based on trying to find the minimum set that could be used to describe the Italian orthography under the assumption that single graphemes generally map onto single phonemes. Based on this strategy, 5 consonant and 9 vowel graphemes that had more than one letter were used (gl gn gh ch sc ia ie io ió iu iá ié iù ii). These graphemes could potentially occur in any place of the graphosyllabic template, and the template was organized into a CCCCVC structure for each of the three possible syllables it could contain. This structure was chosen because there are maximally 4 onset graphemes in Italian (e.g., *Austria* [Austria], which uses the onset /strj/ in the second syllable). One coda consonant was used because, excluding a small number of loan words, only a single grapheme can occur in that position.

A second assumption concerned the coding of geminates. They are represented in the phonology of the database as a single coda consonant followed by a single onset consonant. In the orthography they often correspond to a sequence of two identical letters (e.g., -ss in *casse* [boxes]). Accordingly, these were coded as two single letter graphemes split between the two orthographic syllables. Conversely, when the geminates corresponded to non-identical letter sequences like –gl (e.g., *maglione* [jumper]), these were coded with a single grapheme that was put in the first onset slot of the second syllable which the geminate spanned. Such a distinction is consistent with the conventional splitting of end-of-line words (when the line is out of space) in Italian printed text, which is also explicitly taught to children for handwriting. That is, the geminate letters are split (e.g., *cas-se*, with *se* going to the next line), whereas two consonant letters forming a grapheme are not split (e.g., *ma-glione* is a legitimate split but *mag-lione* is not).

A third assumption that was made was that the semi-vowels /j/ and /w/ were coded by a single grapheme in an onset position. Thus, it was assumed that even if a letter is nominally a vowel, it does not necessarily have to be placed in a vowel position of the template. Rather, it was assumed that a vowel letter may occur in the onset position after a consonant when it is representing a semi-vowel phoneme. Thus, for example, *partiate* [leave], which has the phonology /par.tja.te/, was coded as p(o).a(v).r(c).t(o).i(o).a(v).t(o).e(v) and not p(o).a(v).r(c).t(o).ia(v).t(o).e(v) (o = onset; v = vowel; c = coda). Using these vowels in onset slots of the graphemic template allows only one-grapheme-one-phoneme correspondences to be used.

An alternative to using vowel graphemes in onset positions would have been to use vowel graphemes with two letters (e.g., -ia) including ones that are not necessary with the current coding scheme (e.g., -uo). Apart from having to use many more graphemes, if this strategy was used then, in some cases, a single grapheme would have needed to map to both a vowel and the semi-vowel phoneme. Using vowel letters in onset positions therefore reduces the number of graphemes a great deal and also means that single graphemes map to single phonemes in these cases. For example, the –ia grapheme may either fall in a syllable without a /j/ in the onset associated with it or it may fall in one with a /j/. With the word *partiate* (/par.tja.te/), for example, there is a /j/ after the /t/. With the current coding scheme (p.a.r.t.i.a.t.e), because –i maps to /j/ in a one-to-one fashion, there is no inconsistency. Alternatively, when words without a /j/ are used, such as *angoscia* [anguish] (/angɔʃʃa/), the –ia is not split, and thus there is no ambiguity either.

One advantage of the present coding scheme is that it naturally accounts for context sensitivity, which reflects the fact that the pronunciation of an onset consonant is affected by the vowel that follows it. For example, the letter –g is usually pronounced /g/ when followed by an *a*, *o*, or, *u*, or /ʤ/ when followed by *e* or *i* [30]. This means that in a word like *seguo* [follow] (/segwo/), currently coded as s.e.g.u.o, the –u needs to combine with the –g to activate the correct phoneme. It also needs to activate the /w/ phoneme. The –o is then left to activate the vowel. Alternatively, if the vowel was instead coded as two letters (s.e.g.uo), the –uo would need to perform all three functions – help activate the correct context sensitive onset, activate /w/, and activate a vowel.

### Creating the Training Databases

The graphemic structure of the training database was created in the same way as in Perry et al. [6], where words were first divided into contiguous consonant and vowel sequences. These were then parsed using the longest possible graphemes. To identify cases where vowel letters functioned as semi-vowels, all words where the initial parsing of the graphemes caused there to be less vowel graphemes than vowel phonemes were identified. When this occurred, the onsets of syllables were scanned for /j/ and /w/. If the vowel that came after them started with either an –i or a –u, this vowel was placed in an onset position (N = 7076). Other more minor changes included:

1. the –sc onset was split into –s and –c when it corresponded to /sk/ (e.g., scappa /skappa/; N = 855).
–gl was split as –g and –l when it corresponded to /gl/ (e.g., gloria /glɔrja/ [glory]; N = 836).
3. If there were less orthographic vowels than phonological ones, the string was scanned for all of the multi-letter vowels and the vowel grapheme was split if one was found (N = 813). For example *avvia* /avvia/ [start] has the consonant-vowel sequence [a][v][v][ia], and three phonological vowels. To get the number of orthographic and phonological vowels the same, the –ia was split. Thus, the final graphemes were a.v.v.i.a.
4. The onset –sch was split as –s and –ch (e.g., *scherzo /sketso/* [joke]; N = 329).
5. The vowel sequence –iuo (e.g., *giuoco* /ʤwokɔ/ [play], N = 6), was split as –iu and –o.

This left 191 words which could not be coded, almost all of which were loan words (e.g., *delphi*). Therefore, the final training database contained 63360 words. From these words, the training database for the graphemic parser was constructed by taking each word and creating the set of 3-letter sequences that represented the letters in the attentional window with a grapheme that needed to be parsed at the start (the input patterns). These were paired with the grapheme that occurred at the start of each sequence (the output patterns). See above for an example of this. This meant that there were 417,622 training exemplars. The training database for the TLA network was constructed by simply aligning the graphemes in the graphosyllabic template of the network (the input patterns) and pairing this with the phonology of the words (the output patterns).

### Training the Graphemic Parser

The graphemic parser was created in the same way as Perry et al. [6], where a simple two-layer network with a 3 grapheme memory was trained to select graphemes from the start of strings of letters and also categorize them into onset, vowel, and coda categories. Training was also done in an identical fashion to Perry et al. [6], where different networks were trained on the whole database as well as a number of smaller subsets of words (500, 1000, and 2000 words). The input patterns were the three letter sequences that could be derived so that a grapheme was at the start of each sequence, and the actual graphemes used were those derived from lexical phonology as described above. The output patterns were simply the grapheme and its classification (i.e., onset, vowel, coda). See above for an example of the patterns created for the word *maglione*.

The input layer of the graphemic parser consisted of three main sections that contained 32 letter nodes each (i.e., 31 letters plus one “null” letter). These were designed to represent any possible sequence of letters that could occur in an attentional window that is 3 letters wide. The output layer consisted of all possible graphemes. These were repeated 3 times so any grapheme could potentially be classified into an onset, vowel, or coda category.

### Graphemic Parser Results

There were far fewer errors in Italian than in English – indeed there were only 1001 (.24%) errors for the patterns that were used in training. The errors were not random, which makes it possible to look at the individual types of errors, and these appear in the Materials S1 in File S1.

Whilst the results suggest that the model is not perfect, this is at least in part because there are inconsistencies in the way graphemes are split in Italian, and the errors can help identify predictions that the model makes. For example, *esempii* [examples] and *capii* [I understood] both use an -*ii* letter sequence, but with *esempii*, the -*ii* functions as a semi-vowel and vowel, whereas with *capii* there are two vowels. This type of inconsistency causes the model to make errors with the -ii letter. This means that CDP++ predicts that people will also give a distribution of responses when confronted with the –ii pattern, since it is something which is ambiguous in Italian, and some of the responses will therefore have a different number of syllables to the other ones. Another example of this is the –ia vowel sequence, which can also be parsed into different categories (e.g., *previa* /prevja/ [subject to] (semi-vowel/vowel) vs. *rinvia* /rinvia/ [reject] (vowel/vowel)).

**Figure 3:**
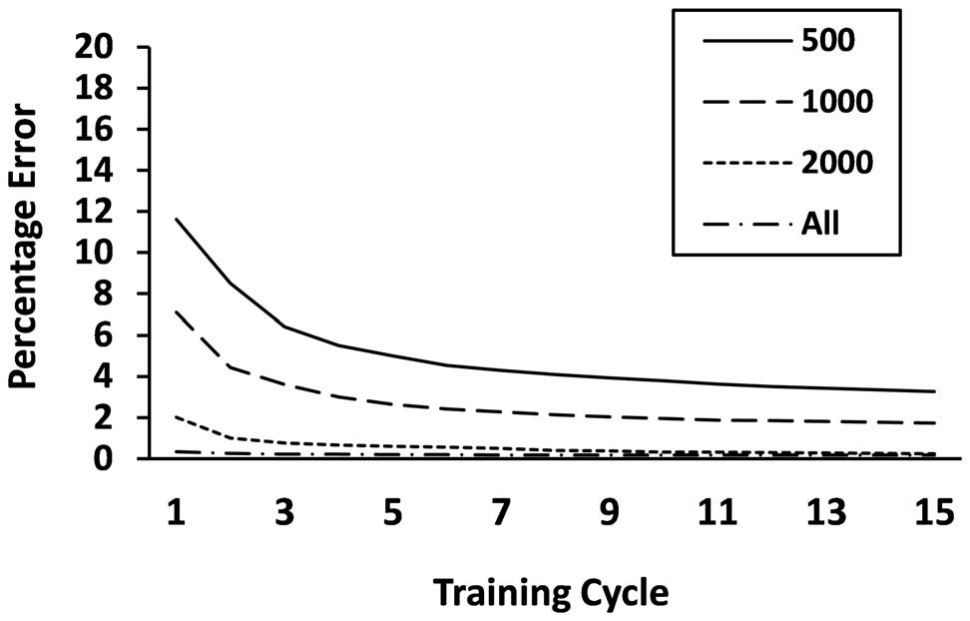
Percentage of graphemes selected incorrectly with networks trained on different numbers of exemplars over 15 cycles of training.

Apart from the results of the fully trained model, the models trained on a small number of exemplars also showed reasonable performance (see Figure 3). Even when the model was trained on only 500 words, it was able to get most correspondences correct. This suggests that choosing the correct graphemes in words in Italian is fairly simple, and can be done with relatively minimal information about the entire database, which might explain in part why reading acquisition is a lot quicker in Italian than in English [31].

### Training the Sublexical (TLA) Network

The TLA network was trained for 20 cycles using the same parameters as in Perry et al. [6]. Phonemes and graphemes were aligned in the same way as in CDP++ (i.e., into syllables).

### CDP++.Italian: Results

The items used for all of the studies simulated below were identical to those in the original studies (The exact results were not reported in a small number of the experiments. When this was the case, we estimated the results from the figures). When words were used that did not exist in the database, they were excluded from the analysis, as were nonwords that were in the database (i.e., nonwords that were actually words). A 3 standard deviation (SD) cut-off was also applied to all of the results, and these items were considered outliers, as were all words that took more than 250 cycles to produce. The number of items removed from the statistics and the reason is reported after each experiment in square brackets. All data sets were run using the same parameter set (see the Materials S2 in File S1) unless otherwise stated. Due to computational constraints, we restricted the lexicon of the model. This was done by only using words in the lexicon that were identical to the one being run, except for the pseudohomophone, neighborhood, and Job et al. [30] simulations, where we used the full lexicon. This was necessary since, with more than 60,000 items in the lexicon, it is very hard to find an optimized parameter set within a reasonable amount of time. In addition, our previous work has shown that whilst examining some properties of feedback in the model is useful, feedback has little impact on data sets that do not need it [4], [8].

### Database Comparisons

The first set of results we examined were those from Barca et al. [29], a database with the reaction times for 625 nouns, 501 of which were in the model's lexicon (most of the others were 4-syllable items). We used a two-step regression analysis to predict the human reaction times. In the first step, we added the onset characteristics of the first phoneme of the words. These were taken from the database of Barca et al. In the second step, we added the naming latencies of the model (in number of cycles). The performance of the model was compared with a number of different regression analyses that used the onset characteristics of the words, log word frequency from the same database which the model used, orthographic neighborhood calculated using Levenshtein Distance [32], number of letters, number of syllables, and word stress. The results showed that the model plus onsets captured slightly less variance compared to the regression equation with all of the terms in it (52.3% versus 53.6%), somewhat more than onsets plus frequency (50.6%), and more than just onsets alone (46.4%). Unfortunately, as can be seen via the difference between the full regression equation and just the onsets, the amount of variance that could be captured above just simple onset characteristics was relatively small (for a similar finding in French, see [11])

### Words, Frequency, and Nonwords

Perhaps the simplest question of all that could be asked about the model is whether it reads aloud real words more quickly than nonwords, and whether high frequency words are read aloud faster than low frequency words. Given that the Italian orthography is very regular, it is conceivably possible that at least the segmental phonology of words could be generated without lexical input, which, ignoring word stress, would predict that nonwords and words would be read aloud at a similar speed. This is not what is found [33], however, and it is been shown that not only are nonwords read aloud more slowly than words, but low frequency words are read aloud more slowly than high frequency ones. This suggests that lexical input is important in Italian reading. Using the same stimuli as Pagliuca et al. [33] where both high and low frequency words were examined as well as nonwords, we examined whether the model would also show this pattern. The results showed that, just like the data, words were read faster than nonwords, *t*(85) = 14.70, *p*<.001, and high frequency words were read faster than low frequency words, *t*(44) = 3.83, *p*<.001 (High Frequency Words: 70.7; Low Frequency Words: 80.4; Nonwords derived from high frequency words: 120.0; Nonwords derived from low frequency words: 125.5) [2 words not in the database, 7 nonwords in the database].

### Stress Regularity/Consistency

**Figure 4:**
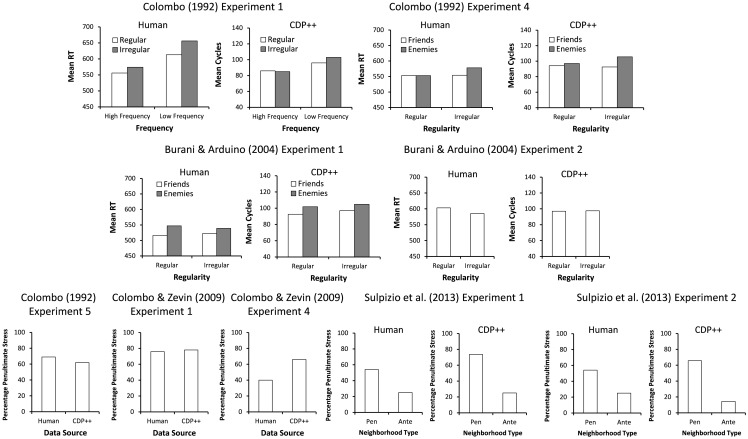
Overall results from the stress regularity/consistency studies. Note: Pen = Penultimate; Ante = Antepenultimate.

Perhaps the results that are the most important in Italian reading are those to do with how reaction times are affected by stress regularity and stress consistency – that is, whether people give slower responses to words with atypical stress due to them not having a possible default stress (regularity – typically assumed to be penultimate in Italian) or due to them having a different stress pattern compared to words with similar spellings (consistency, typically measured as a friends vs. enemies ratio where friends share the same orthographic sequence and phonology but enemies only share the same orthographic sequence). The results of the model on all of the experiments reported below to do with stress regularity and consistency appear in Figure 4.

Colombo [34] ran one of the seminal studies on stress effects in Italian. In her first experiment, she examined stress regularity in both high and low frequency words and found that words with irregular stress were slower to read aloud than words with regular (i.e., penultimate) stress, but that this was restricted to low frequency words. CDP++ showed the same pattern, with a main effect of Frequency, *F*(1, 101) = 75.41, p<.001, Stress Regularity, *F*(1, 101) = 6.46, *p*<.05, and an interaction between the two, *F*(1, 102) = 9.31, *p*<.005. Two t-tests showed that the difference between the high frequency words was not significant, *t*<1, but the difference between the low frequency words was, *t*(47) = 2.88, *p*<.01. (High Frequency Regular: 80.6; High Frequency Irregular: 79.9; Low Frequency Regular: 88.7; Low Frequency Irregular: 96.7). [9 words were not in the database, 1 outlier].

Apart from just stress regularity, Colombo [34] also examined whether other properties of stimuli interacted with the stress regularity (her Experiment 4). She found that stress consistency, which she defined as the extent to which the last 3 letters of a word shared the same stress pattern with other words with the same 3 letters (i.e., stress neighbors), was important. With words that were stress inconsistent (i.e., had more stress enemies than friends), RTs were slower than when they were consistent, but only when the words were also stress irregular. When the words were stress regular, no effect of stress consistency was found. CDP++ showed a relatively similar pattern, with main effects of Stress Consistency, *F*(1, 55) = 9.34, *p*<.005, no effect of Stress Regularity, *F*(1, 55) = 1.32, *p* = .26, which was unlike the data of Colombo where a significant effect was found, and, importantly, an interaction between the two, *F*(1, 55) = 5.17, *p*<.05, which appeared to be caused by the inconsistent words with irregular stress being especially slow (Stress Consistent/Stress Regular: 94.25; Stress Consistent/Stress Irregular: 92.6; Stress Inconsistent/Stress Regular: 97.0; Stress Inconsistent/Stress Irregular: 105.6) [5 words were not in the database].

Burani and Arduino [18] (their Experiment 1) also examined the effect of stress consistency and regularity. They ran an experiment that was similar to that of Colombo [34] where stress consistency was examined, but suggested that their stimuli were better matched than those of Colombo for a number of different reasons (see Burani and Arduino for a list of these). Interestingly, they found effects of stress consistency for both stress regular and stress irregular words. CDP++, showed a main effect of Stress Consistency, *F*(1, 42) = 8.94, p<.01, but not Stress Regularity, *F*(1, 42) = 1.73, *p* = .20, nor an interaction between the two, *F*<1 (Stress Consistent/Stress Regular: 92.6; Stress Consistent/Stress Irregular: 97.1; Stress Inconsistent/Stress Regular: 101.8; Stress Inconsistent/Stress Irregular: 104.8) [15 words were not in the database, many of which were 4 syllables long]. This pattern is therefore very similar to the one reported by Burani and Arduino. The successful simulation of both sets of results suggests that the seemingly inconsistent results between these two studies can potentially be explained once the properties of items are taken into account.

Apart from stress consistency, Burani and Arduino [18] also looked at the overall number of words that share a particular letter sequence, which they called numerosity. They found that stress irregular words with a high numerosity were named faster than stress regular words with a low numerosity. CDP++ predicted a null effect with this dataset, *t*<1 (Regular words: 97.2; Irregular words: 97.6) [8 words were not in the database].

One final study looking at stress neighborhood was run by Sulpizio, Arduino, Paizi, and Burani [35]. They examined the effect of stress neighborhood with nonwords, defined in a similar way to Colombo [34]. With their three-syllable stimuli, they found that having a consistent stress neighborhood had a weak effect on nonwords when the neighborhood favored penultimate stress, but had a strong effect when it favoured antepenultimate stress. They suggested that rather than these results reflecting just stress consistency, as defined by the proportion of stress friends versus enemies, they were likely to occur because of a difference in the numerosity of friends versus enemies. That is, with their nonwords, those in an antepenultimate neighborhood shared a greater number of stress friends and stress enemies than those in a penultimate neighbourhood, even though the consistency ratio was similar for both types of words (for example, a word with 12 friends and 6 enemies has a higher numerosity than a word with 6 friends and 3 enemies, even though both have the same consistency). CDP++ showed a similar pattern to the data, although tended to over-predict the effect of having a consistent stress neighborhood (Experiment 1 (Penultimate vs. Antepenultimate responses), Human: Penultimate Neighborhood: 53% vs. 47%; CDP++: 73% vs. 27%; Antepenultimate Neighborhood: 25% vs. 75%; CDP++: 16% vs. 84%; Experiment 2, Human: Penultimate Neighborhood: 58% vs. 42%; CDP++: 66% vs. 34%; Antepenultimate Neighborhood: 25% vs. 75%; CDP++: 14% vs. 86%) [Experiment 1: 2 nonwords were 4-syllables long].

Apart from studies specifically looking at the comparison between different types of stress dominance, there are also two sets of nonwords run by Colombo and Zevin [36], and hence the proportion of nonwords given penultimate stress can be examined (note that Experiment 1 and 2 in their study used the same nonwords), as well as a set of nonwords used in Colombo [34] (Experiment 5). With the nonword set used by Colombo, 69.8% of nonwords that participants gave reasonable responses to (i.e., were not errors) were given penultimate stress. CDP++ gave a result close to this, giving 61.9% of the stimuli penultimate stress [1 4-syllable nonword]. With the first and second experiment of Colombo and Zevin, the nonwords were deliberately chosen to be biased to give penultimate stress, and this pattern was found with CDP++ and in the real data (CDP++: 78%; Experiment 1: 76%; Experiment 2: 83%). Alternatively, in the fourth Experiment of Colombo and Zevin, a balanced set of items in terms of type of likely stress was chosen, although people produced somewhat more antepenultimate (63.2%) than penultimate responses. CDP++ produced the opposite result, favouring penultimate responses (66.3%) [Experiment 1: 2 outliers; Experiment 4: 1 nonword in the database].

### Orthography-Phonology Consistency

A second type of consistency that can be found in Italian relates to the orthography-phonology mapping. Job, Peressotti, and Cusinato [30] examined this by constructing nonwords that used consonant graphemes that could only be correctly read if the following vowel was taken into account (see the final paragraph in *Graphosyllabic Template* above for the set of vowels that affect some consonants and how these are coded in CDP++). They did this by choosing words with one of these consonants in it, and then constructing two types of nonwords by changing a single vowel in them. In one case, the nonwords kept the same consonant pronunciation as the words they were derived from (the consistent nonwords; e.g., *mercoto* /merkotɔ/, which was derived from mercato /merkatɔ/) whereas in the other case they did not (the inconsistent nonwords; e.g., *merceto* /mertʃetɔ/).

The results of Job et al. [30] showed that when the nonwords were mixed with words, there was an effect of consistency, with the consistent nonwords being read aloud faster than the inconsistent ones, but this did not occur when the nonwords were not mixed with words. CDP++ showed a significant result with this set of items, *t*(46) = 2.12, *p*<.05 (148.1 vs. 164.5 cycles) [1 outlier, 4 Errors, 14 4-syllable nonwords, 1 nonword in lexicon]. To simulate the change of strategy as a function of list composition (i.e., no consistency effect with nonwords only), we reduced the threshold at which phonemes and stress nodes needed to be activated before naming can be finished to .5. This was done because, as noted in Perry et al. [4], it is a reasonable way of simulating lists where only nonwords are used. This is because nonwords tend to generate less activation than words and hence people may reduce their response criterion accordingly when reading blocks of them. With this change, the model did not produce a consistency effect, *t*(49) = 1.42, p = .16 (120.1 vs. 126.1 cycles [2 Errors, 14 4-syllable nonwords, 1 nonword in lexicon]). Job et al. also ran an additional experiment that was the same as the other where the stimuli were run in a nonword only block, except a different set of nonwords were used. The results they found showed no significant difference between the consistent and inconsistent nonwords. CDP++ did not display a significant difference between those two groups either, even using the normal parameters, *t*<1 (150.9 vs. 146.9 cycles) [1 outlier].

A more recent set of experiments that assessed whether words including complex (contextual) print-to-sound rules are named more slowly than words with no contextual rules was run by Burani, Barca, and Ellis [37]. In contrast to Job et al., they examined simple versus complex spelling-sound patterns in words rather than nonwords. In their first experiment, they found that people were slower reading aloud the words with consonants that required a context to predict their phonology correctly. CDP++ predicted the same result, *t*(39) = 2.20, *p*<.05 (89.4 vs. 94.4 cycles) [15 words not in lexicon]. In their second experiment, they added the additional factor of word frequency. Their results were somewhat mixed, presumably because with the complex rule words there was a lower density of complex letter clusters relative to Experiment 1 (See Procedure, Experiment 2), with no effect with high frequency words and the results with low frequency words being much weaker than the previous experiment. This caused the main ANOVA to fail to reach significance by items. The absolute size of the effect with the low frequency words was also smaller than the first experiment (11 ms vs. 24 ms). CDP++ predicted that there should not be a significant effect of consistency with either the high or low frequency items (both *t*'s<1; High Frequency Consistent: 77.4; High Frequency Inconsistent: 78.0 [2 words not in the database]; Low Frequency Consistent: 86.8; Low Frequency Inconsistent: 87.2 [3 words not in the database]).

### Other Effects

Apart from consistency, there are a number of different effects that have been reported in Italian. These include the effect of morphology, pseudohomophony, and orthographic neighborhood. Morphological effects are interesting because CDP++ has no explicit morphological processing layer. Thus, if CDP++ were to capture effects that are presumably due to morphological processing, it would suggest that some of these effects can be explained by factors correlated with morphemic status, such as frequency, rather than some sort of explicit morphological status or the semantics associated with particular morphemes (see e.g., [16]). Burani, Marcolini, De Luca, and Zoccolotti [38], examined this in reading, and they found that a number of different groups, including normal adult readers, read nonwords that were composed of a root and suffix more quickly than morphologically simple nonwords. CDP++ displayed the same result, *t*(29) = 2.40, *p*<.05 (144.5 vs. 158.5 cycles) [1 nonword in lexicon]. Alternatively, when a similar manipulation was examined with words, skilled readers did not show any differences. CDP++ displayed this result also, *t*<1 (93.1 vs. 92.6 cycles) [44 4-syllable words not in the database], although there were a large number of items in the stimuli that it was not able to use. A very similar set of nonwords as Burani et al. [38] was examined by Burani, Marcolini, and Stella [39]. Similar results were also found, except that there was an exceptionally large error rate (21.7%) on the morphologically simple nonwords, which was presumably due to the different composition of the lists the nonwords were part of in the two studies. CDP++ not surprisingly gave very similar results with these nonwords compared to the previous ones, *t*(28) = 2.03, *p* = .052, and also made no errors (144.2 cycles vs. 156.7 cycles; note that one nonword that was actually an exceptionally low frequency word was treated as a nonword rather than removed due to the small number of items and thus the importance of each item in the final significance value). Whilst the difference in the error rate between the model and the real data is interesting, creating errors with CDP++ to try to simulate this aspect of this particular data set is beyond the scope of the current work.

Pseudohomophone effects, where people read aloud nonwords with phonology that sounds like a word faster than nonwords where it does not, are interesting because they are generally believed to show that there is feedback from sublexical phonology in reading (see [4] for a review). Peressotti and Colombo [40] examined this in Italian using pseudohomophones that were orthographically often very strange (e.g., *cjfra*) and similarly matched nonwords, with the idea being that using nonwords with strange sequences of letters meant that none of the effects they found could be due to orthographic similarities between pseudohomophones and the words that they sounded like. They also compared the results to more orthographically normal non-pseudohomophonic nonwords. The results they found showed that the pseudohomophones were read aloud faster than their controls, but the difference between pseudohomophones and the nonwords with more normal spellings was not significant.

Despite the strange orthographic patterns used by Peressotti and Colombo [40], we presented their stimuli to CDP++. Not surprisingly, the model had a high error rate, since it simply could not produce a reasonable answer for some of the nonwords, such as when a –j was used as a vowel. Initial inspection of the results showed that these errors were not distributed evenly across the groups. In the pseudohomophone and nonword control group, the model made 36 and 44 errors, compared to 13 with the orthographically normal nonwords. Because the groups were very large, however (119 items in each cell), we could still examine the RTs from the correct responses. However, rather than using between-group comparisons, as we generally do, we only used within-group comparisons. This is reasonable because stimuli triplets were matched across the groups (e.g., *ansja*, *antja*, and *antia*). The results showed that, like the human data, CDP++ predicted that the pseudohomophones would be read aloud faster than the control nonwords, *t*(57) = 2.52, *p*<.05 (133.0 vs. 143.0 cycles) [80 Errors, 2 outliers]. Unlike the data, however, the pseudohomophones were slower than the orthographically more normal nonwords, *t*(58) = 2.29, *p*<.05 (135.5 vs. 126.2 cycles) [49 Errors, 2 outliers, 9 nonwords in database]. Apart from just the error rates, these results should be taken with great caution because, unlike Perry et al. [5], [6], we allowed the network to run even if a correspondence was very poorly learnt. That is, it triggered many “dead nodes” [5], which we ignored (see [41] for evidence suggesting that nonwords with very strange spellings are not processed by the normal reading system and hence reasonable to ignore). This allowed slower responses in the pseudohomophone and matched nonword control condition that would typically be excluded, and hence is likely to be responsible at least in part for the difference in reaction times between the pseudohomophones and orthographically normal nonwords.

Another way that phonological feedback can be examined was done by Mulatti et al. [42]. They examined nonwords by changing a single letter either in the first position or a latter position of a word (e.g., a word like *serpe* [serpent] was changed to form *berpe* and *babbo* [dad] was changed to form *babro*). They found that nonwords created from a first letter change were slower to read aloud than nonwords created from a latter letter change. They suggested that this was caused by serial processing of sublexical phonology and the way that activation produced at different times interacted with lexical activation. CDP++ was not able to replicate this effect, *t*<1 (124.4 vs. 128.6 cycles). [2 errors, 2 nonwords in lexicon of model]

Finally, neighborhood effects [43], where the effect of words with similar spellings to the one being read aloud is examined, are interesting because they may provide some insight into both learning (e.g., [12]) and the way the lexical system functions (e.g., [43]). In Italian, Arduino and Burani [44] reported that nonwords with many orthographic neighbors (i.e., words that differ by a single letter) were read aloud faster than nonwords with few orthographic neighbors, and that whether the nonwords had a high frequency neighbor or only low frequency ones did not appear to affect the results. When their stimuli were presented to CDP++, there was a significant main effect of whether a nonword had high frequency neighbors, *F*(1, 51) = 4.39, *p*<.05, with the nonwords with high frequency neighbors being named more slowly than those with only low frequency neighbors, as well as a significant interaction, *F*(1, 51) = 5.81 (High Neighborhood/High Frequency Neighbors: 132.8; Low Neighborhood/High Frequency Neighbors: 123.6; High Neighborhood/Low Frequency Neighbors: 116.5; Low Neighborhood/Low Frequency Neighbors: 113.8). [4 errors, 1 nonword in the lexicon of the model]. Two t-tests examining the nonwords with high and low frequency neighbors separately were not significant (high: *t*(25) = 1.60, *p* = .12; low: *t*(26) = 1.86, *p* = .075). It is unclear to us exactly why the model shows the incorrect pattern with the nonwords. However, CDP++ has not been able to simulate neighborhood effects all that well in previous simulation work (e.g., [4], [5]), and thus this may represent a problem with the model. As noted by Pagliuca and Monaghan [12], there might also be a lack of power because there were only 15 items in each cell.

### Acquired Dyslexia

Different types of acquired dyslexia have been reported in Italian, including both surface and phonological dyslexia (e.g., [45]-[47]). One particular area of interest has been word stress (e.g., [21]). In the largest study examining this, Colombo et al. [21] examined 22 patients with Alzheimer's Disease (AD), and classified them into 3 groups based on how advanced their cognitive decline was as measured by the Mini Mental State Examination (MMSE) [48]. They then examined their reading performance on high and low frequency words and nonwords. The words they used were divided into what they called dominant and subordinate stress, with the former defined as those with penultimate stress and the latter defined as those with ante-penultimate stress. The groups were also balanced on stress neighborhood (see [18], [34]) with the dominant words having high stress consistency and the subordinate words low stress consistency.

The results Colombo et al. [21] found showed that the performance of the groups was related to their performance on the MMSE, with the group that scored the lowest also performing the worst. With that group, stress errors accounted for around 35% of all of the errors, and the percentage of correct responses was affected by both frequency and stress type (Dominant stress, High Frequency: 92.2%; Low Frequency, 83.0%; Subordinate stress: High Frequency: 79.2%; Low Frequency: 45.3%). A more complex pattern was found with nonwords, where the most severe group had an average error rate of 25%, and the more severe the AD, the more likely they were to give subordinate stress on the nonwords.

At present, we only simulated the effect of word stress, since simulating the nonword results would have required more than simple parameter changes, which is beyond the scope of this paper. This simplification is reasonable because, whilst phonological deficits and nonword processing problems are often found to co-occur in AD [49], phonological dyslexics have been reported with no obvious phonological processing problems [50], [51]. Caccappolo et al. [50], [51] suggest that this means that sublexical and lexical mechanisms may not be directly linked. In addition, in one of the classic cases on acquired surface dyslexia in Italian [52], a patient was described with “virtually normal” (p. 283) performance when reading nonwords, but made many stress errors reading some types of words.

To simulate the word results of Colombo et al. [21], we simply used the same strategy for simulating surface dyslexia we have used elsewhere [4]–[6]. We did this by increasing the frequency scaling of the lexicons to .75, with the idea that this simulates additional difficulties in lexical access. We also reduced the amount of activation going into the phoneme output buffer by changing the excitation and inhibition parameters from the phonological lexicon to the phoneme output buffer to .03 and −0.03. For the sake of simplicity, we did not reduce the level of activation going to the stress output nodes under the assumption that the generation of phonemes in AD is more difficult than the generation of stress information. Obviously, in the future, it would be possible to examine the effect of reducing activation to both phoneme and stress output nodes should the data dictate it.

With the parameter changes noted, CDP++ produced results very similar to those of the severe group on overall error rates (Correct %, Dominant stress, High Frequency: 91.2%; Low Frequency: 77.1%; Subordinate stress, High Frequency: 82.9%; Low Frequency: 54.3%). The distribution of errors was also very similar, with 44% of the errors due to stress and 56% coming from other sources. These results suggest that the effects of frequency and stress dominance are inherent properties that the model is sensitive to, and that when it is parameterized such that it does not perform at near perfect accuracy, the most likely items it makes errors on are also the most likely ones that people do after cognitive decline due to AD.

### Priming

All of the previous simulations relied on getting the model to produce output in a simple naming task, with each item run entirely independently of the others. However, there is also some data on stress priming in Italian, where the effect of being primed with a word that has the same or different stress to the one being named has been examined. At present we will not try to simulate all aspects of priming, as there are a number of non-trivial issues that would need to be considered to do this. These include how decay in representations should be set (i.e., the amount activation in representations reduces from one word to the next), how primes should be treated when a second word appears on top of them, and how to implement aspects of the prosodic processes not currently implemented, such as how stress is stored in the linguistic system over time.

Despite the problems of modelling priming, the type of results the model would predict ignoring more intricate matters can be examined. In terms of simple priming where a prime precedes a target word, Sulpizio, Job, and Burani [53] found that when a prime was presented for 83ms before a target with the same stress pattern, the target word was named faster than if the preceding word had a different stress pattern. They found that this occurred irrespectively of whether the target word had penultimate or antepenultimate stress. Our explanation is the same as offered by Sulpizio et al., which is that this may be explicable via the pre-activation of stress information (stress nodes in the model), which would then either reach threshold faster if the stress information is congruent or more slowly if it is incongruent. To examine this, we ran the model using the words of Sulpizio et al. with a reduced stress criterion (.58 instead of .68), which simulates the ability of the model to reach the stress threshold faster. The results showed that the size of the reaction time differences between the model with the normal and low stress criterion were relatively similar across the words with penultimate and antepenultimate stress (Penultimate Stress, Normal/Low, 99.9, 94.6; Antepenultimate Stress, Normal/Low: 106.3, 98.6; Priming effect: Penultimate: 5.3; Antepenultimate: 7.7) [9 words not in lexicon]. Note that due to the repeated nature of the comparison and the fact that the model is entirely deterministic, statistics are not reported since even the smallest of priming effects are almost always significant.

Apart from standard priming, Colombo and Zevin [36] examined the effect of priming across a number of trials. In particular, they used a paradigm where a set of words or nonwords with the same stress would occur before a target word and the effect of the prime words examined. The results they found suggested that the main change caused by the prime words and nonwords was a change in the dominance between lexical and sublexical processing, with people making more errors on words that did not have a dominant stress pattern when the primes caused more sublexical processing. To simulate this, we ran the words of Colombo and Zevin's first experiment, where they examined the effect of nonwords typically given penultimate stress on words with antepenultimate stress. With the normal parameter set, the model makes no stress errors. To simulate a change of dominance between lexical and sublexical routes, we increased the excitation strength of the TLA to phoneme output and stress output buffer to .12, and reduced the strength of the parameters from the phonological lexicon to the stress output buffer by .02. The results showed that this increased the error rate on the words to 14.3%, which is very similar to the experiment of Colombo and Zevin. Obviously, there are many ways we could have changed the balance between the two routes, but the results here show that a change of balance is likely to cause stress errors in the way Colombo and Zevin predicted.

### The Role of Sublexical and Lexical Process

An important facet of the results that we have not explored is to what extent the lexical and sublexical parts of the model are responsible for the results. One way to examine this is to look at the performance of the model without sublexical or lexical input. This isolates the extent to which the results are simply caused by one route or the other. We first did this on the large database we first examined [29], removing all sublexical input. This caused the amount of explained variance to drop from 52.3% to 49.4% (note that just onsets alone account for 46.4% of the variance and the correlations without onset coding are *r* = .39 for the model with sublexical phonology and *r* = .24 for the model without sublexical phonology). We then examined all of the stress consistency studies without sublexical phonology. None of them even produced a trend towards a significant stress consistency effect, and nor was there an orthography-phonology consistency effect with Burani et al.'s [37] words. Next, we removed lexical activation from the pseudohomophone simulations of Peressotti and Colombo [40], and there was no longer a pseudohomophone effect (*t*<1). These results basically suggest two things. First, that phonology plays an important role in the quantitative performance of the model, as it does in other versions of CDP (e.g., [4]). Second, that stress consistency effects are caused by the interaction of lexical and sublexical processing and that pseudohomophone effects are caused by feedback from sublexical to lexical phonology and back again.

### Inconsistent Findings

Whilst the model produced reasonable results across a broad spectrum of experiments, there were a number of results that the model produced that were qualitatively different to the human ones that were not discussed. These include (a) no significant difference between nonwords created by changing one letter at the start of a word compared to the end of a word as reported in Mulatti et al. [42]; (b) no stress regularity effect in Experiment 4 of Colombo [34]; (c) no significant difference between the high numerosity irregular and low numerosity regular words in Experiment 2 of Burani and Arduino [18]; and (d) no significant difference with the low frequency words with complex versus simple rules in the Experiment 2 of Burani et al. [37].

Whilst we have no definitive explanation for why the model did not capture these, in all cases, the absolute size of the effect reported in the studies was small. In Mulatti et al. [42] it was 15 ms, in Colombo (Experiment 4) [34] it was 13 ms, in Burani and Arduino (Experiment 2) [18] it was 18ms, and in Burani et al. (Experiment 2) [37] it was 11 ms. Alternatively, the size of the effects in all of the experiments where the model did find a significant result, excluding the frequency effect reported in Pagliuca et al. [33], was larger (Colombo [34] (Experiment 1): 43 ms; Colombo [34] (Experiment 4): 24 ms; Burani and Arduino [18] (Experiment 1): 24 ms; Job et al. [30] (Experiment 1): 21 ms; Burani et al. [37] (Experiment 1): 24 ms; Burani et al. [38]: 48 ms; Peressotti & Colombo [21]: 35 ms). Given this, it suggests that it would be worthwhile investigating ways to make the model more sensitive to smaller effects in the future.

Actually finding ways to increase the sensitivity of the model may be particularly challenging, especially for the nonwords of Mulatti et al. [42]. This is because, even though one group of their nonwords differed from their basewords only on the first letter, they often shared start sequences with many other words, and hence their uniqueness to any other words based on serial position may not be as much as the examples in the title of their article might suggest (*zeading* vs. *reazing*). For example, the first nonword reported in their stimuli set, *berpe*, differs in the first letter compared to the baseword *serpe* from which it was created. However, it only differs in the 4^th^ letter with *berci* [yell] (there are in fact 102 other words that start with *ber*). This can be compared with the control *babro*, which differs in the 4^th^ letter compared to its baseword (*babbo*). This means that any early effects of phonological feedback generated serially would activate *berci* and *babbo* to a similar amount, and thus a positive feedback loop from these words being activated should help both nonwords similarly. The main difference then is that *babbo* is a closer neighbour to *babro* than *berci* is to *berpe* (one vs. two letters different). This means that, after the 5^th^ letter is parsed and activation generated, *babro* is likely to be activated more than *berci* since two phonemes would differ from the nonword compared to one. Such fine differences may be very hard for computational models such as CDP++ to capture via a lexical feedback loop.

## Discussion and Conclusions

The present simulations show that CDP++ did a reasonable job predicting many of the different data patterns that have been reported in the literature. The two most important effects have to do with stress and orthography-phonology regularity/consistency. Stress regularity/consistency is important for the development of a comprehensive model of reading aloud but these effects have received little attention in languages other than Italian (but see [19], [54]), probably because most modelling studies have focused on monosyllables (but see e.g., [6], [19], [55]). Orthography-phonology regularity/consistency is important because, historically, it has been the crucial benchmark effect that challenged rule-based models (e.g., [56]) in favour of connectionist models (e.g., [4]). For both of these theoretically important effects, whilst not perfect, CDP++ has captured the data remarkably well.

The ability of CDP++ to simulate various aspects of stress in Italian suggests that the mapping of orthography onto stress nodes, as implemented in the Italian and English CDP++ [5] model, is a powerful and general mechanism that does not seem to be specific or restricted to a given language. The ability of the model to simulate consistency effects in Italian provides yet another demonstration that the CDP family of models is highly sensitive to consistency, as has been shown by the English model on a number of large and exceptionally well-controlled data sets (e.g., [57]). Together, this suggests that graded consistency effects are likely to be an inherent property of the type of network and learning algorithm used, and not something that is specific to a particular orthography.

It is worthwhile comparing the results of CDP++ to those of the PDP model of Pagliuca and Monaghan [12]. Our model differs from theirs in a number of important ways. In particular, we used a lexical route under the assumption that the sublexical route cannot learn all relationships between orthography and phonology. Thus, at least when reading words, our model can perform essentially flawlessly. Alternatively, the model of Pagliuca and Monaghan was only able to read 93.7% of words correctly. It seems likely that if the network of Pagliuca and Monaghan was trained for longer or with a more powerful algorithm, better accuracy could probably be obtained. However, whether the model would still capture nonword stress consistency effects with additional training would need to be explored.

When comparing the two models on the results of the experiments described above, it also becomes clear that CDP++ provides a better fit of the available empirical data than the PDP model of Pagliuca and Monaghan [12]. CDP++ was able to correctly simulate all of the results that were correctly simulated by Pagliuca and Monaghan as well as many others that Pagliuca and Monaghan did not examine. There were also effects that were correctly simulated by CDP++ but not Pagliuca and Monaghan's model (e.g., the effect of stress consistency effect using Burani and Arduino's [18] items).

Given there are some similarities between the models, one might try to isolate why CDP++ performs better than Pagliuca and Monaghan's [12] model. One possibility is that CDP++ uses graphemes and not letters in the input layer. However, given the simplicity of the Italian orthography, and given that Pagliuca and Monaghan organized the representations of their model into a syllable structure (as we did with CDP++) which allowed their model to generalize to nonwords very well, the effect that graphemes have over simply letters may not be especially large. Given this, the other alternative is that the relationships people learn between spelling and sound and spelling and stress are relatively simple, and are hence approximated well via a linear network. This would mean that using a 3-layer network that allows complex and more specific non-linear relationships to be learnt may allow the network to learn things that people do not (see Perry et al. [11] for a further discussion about this in terms of the French orthography). It also means that knowing whether a PDP model trained to be almost perfect on words would behave similarly to the current model of Pagliuca and Monaghan or whether it would learn additional non-linear relationships is important. In this case, additional learning to improve the overall performance of the model on words might also cause it to over-fit the data and hence learn more complex relationships that people do not.

In addition to nonword consistency and stress regularity, we also investigated pseudohomophone, morphology, and neighborhood effects. CDP++ was able to produce a pseudohomophone effect, and, like Pagliuca and Monaghan's model, it captured a morphological effect but failed to capture the full pattern of neighborhood effects in Arduino and Burani [44]. The pseudohomophone effect is interesting as it has always been difficult for PDP models to simulate this class of effects, and has generated a reasonable amount of debate (see [4] for a discussion). The morphological effect with nonwords confirms that both CDP++ and the model of Pagliuca and Monaghan are sensitive to morphology even though they do not have morphological processing layers. CDP++ also showed that, like the real data, the reaction times it produces with words were not affected by morphology. Finally, with the neighborhood effect, Pagliuca and Monaghan showed that their model was sensitive to this variable. However, they used a larger and currently untested stimuli set, and they also suggested that different versions of their network might be differentially sensitive to this. Obviously, a mega-study of Italian words would be useful for investigating these effects further.

Apart from simulating data of normal readers, we also investigated data from acquired dyslexia. Whilst we did not try to model all of the patterns that exist, we did show that, with two very simple parameter changes, CDP++ can produce a stress dominance effect that is of a similar level to the group of patients that produced the largest effect in Colombo et al. [21] – that is, it showed the most errors on low frequency words with subordinate stress. The model also produced an overall error rate that was very similar to that group. Whether a PDP model is able to approximate this is currently unknown, and represents an interesting challenge given that simulating surface dyslexia has historically been a problem for such models (see e.g., [58]).

An important added value of the present modelling enterprise is the fact that CDP++ was able to simulate seemingly discrepant findings, where conflicting results have been reported using essentially the same manipulation. One of the most disconcerting discrepancies was the one between the results reported by Colombo [34] and Burani and Arduino [18] with respect to stress regularity/consistency, with Burani and Arduino suggesting that the difference may be due to the items that were used. CDP++ correctly simulated both sets of results, which shows that their discrepant findings may indeed be due to the actual items selected.

Finally, the model was also able to simulate quite complex findings that depended on list context manipulations (see also [4]). For example, in Job et al.'s [30] first two experiments, the authors found a nonword consistency effect in mixed lists of words and nonwords but not in pure lists of only nonwords. They suggested that this occurred because nonword reading can benefit from lexical feedback, and modulating the proportion of nonwords affects the extent of this lexical influence.

Our suggestion, alternatively, is that modulating the proportion of nonwords affects people's response criterion (i.e., when they are willing to name the word), and this produces in the model the same pattern observed in the human data and is also consistent with other strategic manipulations that have been reported [4].

In summary, the present work has shown that CDP++ can be easily transposed to a regular orthography with a fairly complex stress system, including mechanisms to do with grapheme parsing and learning. The model is available on-line and can be used to predict results before actually running the critical experiments.

## Supporting Information

File S1 Contains the following files: **Materials S1. Materials S2.**
(DOCX)
