# Supplementary material for: CDP++.Italian: Modelling Sublexical and Supralexical Inconsistency in a Shallow Orthography
Source: PLoS One. 2014 Apr 16;9(4):e94291. doi: 10.1371/journal.pone.0094291 (PMC3989230; doi:10.1371/journal.pone.0094291)
Supplement: File S1 — Contains the following files: Materials S1. Materials S2. (DOCX) [file pone.0094291.s001.docx]

Supporting Materials S1

Types of errors the graphemic parser made. The number in brackets represents the number of times the error occurred.

(552) Correct: i (onset); Error: i (vowel), e.g., *briosi* /brjɔzi/ [lively]

(243) Correct: i (vowel); Error: i (onset), e.g., *diurni* /diurni/ [daytimes]

(61) Correct: s (onset); Error sc (onset), e.g., riscrivo /riskrivɔ/ [write back]

(51) Correct: consonant grapheme; Error: consonant grapheme in different category, e.g., *sinistr* /sinister/, where the second –s was a coda rather than an onset (these were generally loan words that would be unknown to most Italians)

(22) Correct: u (vowel); Error: u (onset), e.g., *langua* /laɲgua/ [languish]

(19) Correct: grapheme with two letters starting with i (e.g., -iù); Error: -i grapheme selected, e.g., giù‎ /gu/ [below]

(19) Correct: u (onset); Error u (vowel), e.g., *santuari* /santwari/ [sanctuary] (these tended to be obscure words)

(12) Correct: gl (onset); Error g (onset), e.g., gliela /ʎela/ [a pronoun for double objects]

(11) Correct: i (vowel); Error: grapheme with more than one letter selected e.g., *magia* /maʤia/ [magic]

(9) Correct: sc (coda); Error: s selected, e.g., *kovatsch /*kovaʃ/ (these were obscure loan words with no obvious Italian-like parsings)

(2) Correct: iu (onset); Error: iu (vowel), e.g., *caciuola* /kaʃwɔla/ (these were generally obscure low frequency words)

Supporting Materials S2

Parameters used in the model.

Lexical Route

Features

Feature to letter excitation: .005

Feature to letter inhibition: -.8

Letters

Letter to letter inhibition: -.3

Letter to orthography excitation: .047

Letter to orthography inhibition: -1.0

Orthographic lexicon

Orthography to orthography inhibition: -.1

Orthography to letter inhibition: 0

Orthography to phonology excitation: 1.9

Orthography to letter excitation: 0

Phonological lexicon

Phonology to phonology inhibition: -.12

Phonology to phoneme excitation: .135

Phonology to phoneme inhibition: -.135

Phonology to orthography excitation: 1.5

Phoneme Output Buffer

Phoneme to phoneme inhibition: -.002

Phoneme to phonology excitation: .07

Phoneme to phonology inhibition: -.16

Sublexical Route

Graphemic parsing cycles per letter: 10

Sublexical network to phoneme output buffer/stress output node activation: .08

Level of activation which a letter must be over before graphemic parsing begins: .2

Temperature (s) in the sublexical network: 3

Learning rate (e) in the sublexical network: .05

Dead node level: 0

Word stress parameters

Stress node naming criterion: .65

Phonological lexicon to stress output node excitation: .08

Phonological lexicon to stress output node inhibition: -.055

Stress output node to stress output node lateral inhibition: -.038

Overall parameters

Overall activation rate: .25

Lexicon frequency scaling: .5 * (log (word frequency + 2)/log (maximum word frequency + 2))

Phoneme naming activation criterion: .7

Cycle-to-cycle stopping criterion: 0.0023
